# Cannabis Use Disorder Impairs Motor Cortical Plasticity

**DOI:** 10.3389/fpsyt.2020.589716

**Published:** 2020-11-04

**Authors:** Xiaoli Liu, Xueming Xu, Liyun Deng, Shaochang Wu, Dongsheng Zhou, Wanbo Lu

**Affiliations:** ^1^Ningbo Key Laboratory of Sleep Medicine, Ningbo Kangning Hospital, Ningbo, China; ^2^Department of Psychiatry, Taizhou Second People's Hospital, Taizhou, China; ^3^Department of Neurology, Lishui Second People's Hospital, Lishui, China

**Keywords:** cannabis use disorder, intermittent TBS, cortical plasticity, pair-pulse short-interval intracortical inhibition, transcranial magnetic stimulation

Following the legalization of cannabis in many countries, more and more people have begun to take cannabis recreationally. However, as with methamphetamine and heroin, cannabis is an addictive substance, and long-term use can lead to compulsive drug-seeking behavior, changes in brain morphology (1) and function (2), and an increase in the risk of mental illness. It is estimated 20% of cannabis-users meet the criteria of DSV-5 cannabis use disorder (CUD) (3).

For better CUD treatment, it is important to understand the neural mechanisms of cannabinoids inducing long-term alteration in brain function and how to lead to compulsive drug-seeking behaviors, but these have not been clarified. In animal researches, it has been suggested that repeated exposure to cannabis in rat can have detrimental effects for synaptic plasticity and then result in long-term alteration in synaptic efficacy. It remains unknown that whether the synaptic plasticity of cannabis abusers is impaired. Exploring the plasticity in CUD patients can provide more evidence at mechanism level for further study and CUD treatment. Transcranial magnetic stimulation (TMS) as a safe and non-invasive neuromodulation technique can be used not only to modulate excitability and inhibition of the brain cortex to treat diseases, but also to quantify the degree of long-term potentiation (LTP)-like and long-term depression (LTD)-like of cortical plasticity, with combination of electromyography (EMG). Previous studies found the impairments of cortical plasticity of primary motor cortex (M1) in people addicted to heroin (4) and methamphetamine (5). Therefore, we raise the questions of whether the cortical plasticity of CUD patients is impaired and, if so, how the severity of drug addiction correlates with impairment of cortical plasticity.

Recently, a study of TMS has explored the cortical plasticity of M1 in CUD patients (6). The study enrolled 45 young subjects aged 20–30 years old, including 15 healthy subjects, 15 subjects diagnosed with CUD, and 15 long-term cannabis users not diagnosed with CUD. Three TMS protocols were used in the study: theta burst stimulation (TBS), pair-pulse short-interval intracortical inhibition (SICI), and single pulse TMS. TBS is one of repetitive TMS (rTMS) intervention. It can induce short-term or long-term changes in corticospinal excitability. One pattern of TBS is continuous TBS (cTBS), which is used to inhibit synaptic plasticity; the other is intermittent TBS (iTBS), which is used to increase synaptic plasticity (7). The single pulse protocol involved delivering 20 pulses with an intensity adjusted to the test stimulus (TEST) (the intensity of TEST can evoke about 1 mV MEP) to measure cortical excitability as MEP baseline (8). SICI was described as randomly delivering an 80% active motor threshold (AMT) conditioning stimulus (CS) (*N* = 10) and a pair-pulse CS&TEST (*N* = 10) with inter-stimulus interval of 2 s (9). The experimental procedure was as follows: the baseline measurements were performed including baseline MEP, SICI MEP, severity of cannabis dependence assessed by Cannabis Use Disorder Identification Test-Revised and the estimated elapsed time since last cannabis exposure measured by the concentration ratio of 11-nor-9-carboxy-tetrahydrocannabinol (THCCOOH) to tetrahydrocannabinol (THC) in plasma. Then each subject received one session of cTBS and iTBS intervention (1-week interval); post-treatment evaluation was performed at four points in time (0–3/6–9/12–15/18–21 min).

The outcomes showed that, before intervention, the ratio of SICI MEP to TEST amplitude in both cannabis-taking groups was significantly lower than in healthy subjects, and the ratio of SICI MEP to TEST amplitude was positively correlated with THC level in cannabis users. Although there was no difference in the ratio between cannabis users and non-CUD users after excitatory iTBS intervention, the ratio of MEP baseline of CUD users was higher than healthy and non-CUD users after inhibitory cTBS intervention.

This study shows that the CUD's cortical plasticity was impaired. The baseline results showed that the motor cortex inhibition of both cannabis group was inhibited insufficiently under the inhibitory SICI protocol. Furthermore, CUD group showed a less pronounced response to inhibitory cTBS intervention than the other groups. Therefore, we conclude that regular long-term cannabis abuse could damage to the plasticity of the motor cortex. In addition, this study also addressed the relationship between the severity of addiction and the impairments of cortical plasticity. The more severe the cannabis dependence, the less pronounced the response to cTBS intervention in CUD. The study also found that the less inhibition under the GABAA-mediated SICI protocol the higher of the THC level. The primary target-mediating THC's characteristic psychoactive and reinforcing properties is the cannabinoid 1 receptor (CB1R), and it is mainly expressed on GABA inhibitory neurons. Because it is an agonist of CB1R, THC can activate this receptor and suppress GABA neurons (10). Maybe we can determine the correlation between SICI and plasma THC level from this perspective.

The study was a cross-sectional study that reported that cannabis use disorder impairs motor cortical plasticity, but there is the possibility that subjects with impaired motor cortical plasticity are more vulnerable to CUD. So, Cross-sectional, and longitudinal data is required to could further illustrate the association the between CUD and motor cortical plasticity clearly. The study was performed to quantify the synaptic plasticity of the motor cortex through the amplitude of MEP. However, MEP may also indicate the excitability of the output neuron subgroup (11). We can find more neurophysiological indicators, for example, resting EEG power or the combination of TMS and electroencephalogram (TMS-EEG), to support the research conclusions. Researches related to addiction commonly focused on drug-induced alterations in subcortical regions (e.g., ventral striatum, hippocampus, amygdala) and the prefrontal cortex (12). M1 is not the brain area of mainstream concern, and it is difficult to explain the mechanism underlying the formation of addiction. But brain imaging studies found that cocaine or nicotine cue-induced activation of the sensory association cortex and motor cortex could predict relapse or craving status of these subjects. This study also inspired future research to explore the relationship between plastic injury of CUD motor cortex and motor function.

## Author Contributions

XL, XX, and LD wrote this manuscript. SW, DZ, and WL revised this manuscript. All authors contributed to the article and approved the submitted version.

## Conflict of Interest

The authors declare that the research was conducted in the absence of any commercial or financial relationships that could be construed as a potential conflict of interest.
